# Cultivating Coaching Excellence: Cementing Skills through Practice and Feedback with a Realistic OSTE

**DOI:** 10.1007/s40670-025-02614-2

**Published:** 2026-01-17

**Authors:** Jessica Servey, Hannah G. Kleber, Irene Chi, Erin S. Barry

**Affiliations:** Uniformed Services University, 4301 Jones Bridge Rd, Bethesda, MD USA

**Keywords:** Faculty development, Simulation-based education, Coaching in medical education, Objective structured teaching encounter (OSTE), Competency-based medical education (CBME), Academic coaching

## Abstract

As competency-based medical education (CBME) expands, faculty are increasingly expected to coach learners in goal setting, reflection, and self-directed learning. This adjustment from previous educational styles is a necessary change to enhance learner engagement and subject retention, however, scalable and standardized approaches to developing faculty coaching skills remain limited. Simulation-based strategies, such as the Objective Structured Teaching Encounter (OSTE), offer opportunities for deliberate practice and feedback, yet their application to faculty coaching development is underexplored. This study describes the design, implementation, and early outcomes of a four-case OSTE developed to prepare faculty for longitudinal academic coaching roles in undergraduate medical education (UME). Fifty-three faculty members across three cohorts participated in a four-hour OSTE consisting of standardized student encounters aligned with required coaching sessions in the curriculum. Each faculty member completed one coaching encounter after which they received structured feedback from standardized students, peers, and master coaches and these faculty observed three other encounters and provided their own feedback following the observations. Data sources included immediate written feedback forms, follow-up surveys, and observation checklists assessing five foundational coaching behaviors; descriptive statistics and trend review of comments were used to summarize findings. Faculty frequently demonstrated key coaching behaviors, including listening without interruption (90.3%) and eliciting student goals (77.4%). Participants reported increased confidence and preparedness for coaching, with 83.8% agreeing the simulation aided their preparation and 78.4% finding the cases realistic. Qualitative comments reflected recurring themes of hands-on practice, realism of standardized scenarios, and increased comfort applying coaching skills. The OSTE provided a feasible, no cost, and psychologically safe method for practicing foundational coaching skills before faculty engaged in longitudinal coaching relationships. This study introduces a novel application of simulation-based education to develop academic coaches and highlights OSTEs as a practical strategy to support coaching readiness within CBME-aligned UME programs.

## Introduction

Educational strategies in medical education continue to evolve in response to learner needs and the broader shift toward competency-based medical education (CBME). As programs move away from the “sage on the stage” approach and place greater emphasis on self-directed learning, reflective practice, and individualized goal setting, coaching has emerged as a key approach for supporting learner development. Coaching has gained recognition as a transformative approach to professional development across various industries and is making its way into healthcare. Coaching is defined by the International Coaching Federation as “partnering with clients in a thought-provoking and creative process that inspires them to maximize their personal and professional potential” [1]. Coaching has been associated with improvements in communication, leadership, and lifelong learning skills and has been increasingly integrated into undergraduate medical education (UME) to strengthen students’ ability to set goals and make meaning of their learning experiences [1–9].

Despite the growing emphasis on coaching within medical education, training faculty members to become effective coaches remains a challenge. Globally, coach training varies widely in terms of curriculum, cost, assessment, time required for training, and delivery. Some programs rely on mentorship models, while others incorporate formal certifications or structured faculty development initiatives. However, a standardized curricular approach to coach training, specifically within health professions education is lacking. Logistical and structural challenges further complicate the incorporation of faculty development in coaching. Many established coaching certification programs require significant financial investment and extensive time commitments, which can be prohibitive for faculty balancing multiple academic and clinical responsibilities. Additionally, integrating faculty coaching development into existing medical education curricula is often constrained by competing priorities, such as accreditation requirements, clinical teaching demands, and limited institutional resources. Depending on what the coach is needed for, these may outweigh the benefits of sending a faculty member to a formal coaching training engagement. Another barrier is the transition from an evaluator role to a coaching role, requiring faculty to build trust, ensure psychological safety, and shift toward a growth-oriented feedback model [6, 7, 10]. Often with the above challenges faculty development for academic coaches is superficial, individual readings, or attended if individual scheduling permits assuming clinical educators will learn the skills automatically.

Given these challenges, institutions require feasible and scalable approaches to help faculty practice new skills to shift from a traditional advising role to adopting a coaching mindset [11]. Simulation-based education has long been recognized as an effective strategy for skill acquisition in clinical training, yet its application to faculty coaching development remains underutilized. The Objective Structured Teaching Evaluation, or alternatively Evaluation, (OSTE) - first described in 1992 [12] - is one such simulation-based approach that provides a controlled, psychologically safe environment in which faculty can practice coaching interactions with standardized learners, receive structured feedback, and refine their skills [13, 14]. OSTEs have been used across a variety of health professions and educational levels, including in dentistry [15], pharmacy [16], nursing [17], and UME [5, 18–21], resident [22, 23], and faculty across multiple specialties [5]. Originally developed to strengthen general teaching and feedback skills [13], OSTEs have since been applied to numerous domains such as procedural instruction [22, 24], ethics [25], professionalism [26], leadership [23], and clinical behavior [27].

Best practices for OSTE design highlight the importance of deliberate practice, structured feedback, role clarity, and learner reflection [16, 28]. These principles align closely with the core tenets of coaching, making OSTEs a promising, yet underutilized, tool for coaching development. While OSTEs have been extensively employed to enhance teaching effectiveness, their application to faculty coaching competency development, particularly within longitudinal UME coaching programs, remains largely unexplored. This gap is notable given the increasing expectations for faculty to support learner goal setting, progress monitoring, and self-regulated learning within CBME frameworks. Faculty require opportunities not only to learn coaching principles, but also to rehearse those skills in realistic interactions with guided feedback before using them with students.

To address this need, we implemented a four-case OSTE as part of a longitudinal academic coaching program for UME faculty. By integrating standardized student interactions, structured observation, and multi-source feedback, the OSTE was designed to strengthen foundational coaching behaviors while providing a psychologically safe environment for practice. The purpose of this study was to describe the design, implementation, and early outcomes of an OSTE-based faculty development model for academic coaching in UME. Specifically, we asked: What can be learned from the implementation of a multi-case OSTE designed to prepare faculty for longitudinal academic coaching roles?

## Materials and Methods

### Context

Our medical school is a large medical school with 170 to 180 medical students matriculating annually. In 2022 we initiated a longitudinal coaching program where one faculty coach would facilitate intentionally placed, required coaching sessions (either individual or group sessions) with each medical student (each coach was assigned eight students) over a four-year period. The coaches were either physicians or PhD educators who had faculty appointments in the School of Medicine at the university. The coaches are required to have a minimum number of teaching contact hours within the curriculum. There are 12 required scheduled coaching sessions during the four years, each one at specific points in the student’s education. These include two required group sessions and ten individual sessions. While there are specific academic areas to address at each coaching session, conversations can be adapted by the student (the coachee) based on their own needs and desires. We developed an intentional faculty development plan for each of these groups of coaches [29] including an OSTE. DBS.2022.371, was reviewed by the USUHS Human Research Protection Program and determined to be program evaluation, exempt from review by the Institutional Review Board.

### OSTE Development and Training

Faculty members who were training to become a longitudinal coach participated in the four case OSTE as part of their academic coach training. All standardized students recruited were in the last 18 months of their education or they were students on some type of administrative hold from the curriculum. The cases were developed using real and true to life examples as a foundation. The details were changed to meet the learning and practice objectives of each case. Each case was aligned with a specific required coaching session in our longitudinal coaching program to make the OSTE more realistic. The cases consisted of a role for the faculty member, a role for the standardized student, and additional paper “dashboards” to mimic the digital assessment dashboards utilized at our school. These dashboards contain academic and other data indicating where the student is aligning in their medical school journey with graduation and other requirements [28]. These cases were written by one faculty member and reviewed by others for realism. The simulation roles may have included information regarding three months to two and a half years of a simulated relationship between the faculty member and the standardized student. Students were trained for sixty minutes in the overall logistics of the simulation, script review, objectives of each individual case, and delivery of feedback to the faculty member using first-person communication as noted for training of standardized patients [30]. Standardized students were given a list of faculty and could report any conflict of interest or any discomfort they may have if they needed to deliver constructive feedback to a specific faculty member. During the discussion portion of the training session, standardized students also gave feedback from their perspective regarding the realism of the case to ensure authenticity.

There was an individual faculty who would be overseeing a single OSTE room, each of whom had formal International Coaching Federation (ICF) training and certification or experience administering coaching programs at another medical school who will be termed “master coaches” in the manuscript. These master coaches trained 60 to 90 min annually prior to the simulation. Their training included an overview of the objectives of each case, logistics of the entire simulation, how the training of the standardized students occurred, and reinforcing that the simulation was about practicing coaching principles and synthesis of all of the prior information received in the faculty development. In the first year, four of the master coaches were involved in coaching programs at other undergraduate medical schools. They also gave feedback regarding the entire faculty development process to train our coaches. The second two years, a small cohort of master coaches oversaw the rooms and were involved both years. During the second year using a standard setting approach [31] the master coaches discussed and agreed upon five behaviors as essential foundational skills for coaches. The group, who also performed the didactic training, felt all the trained faculty longitudinal coaches could demonstrate these five skills after only six hours of specific coaching training. These checklists were dichotomous behavior checklists and were completed by the master coach in each of the simulation rooms. These were created as part of a program evaluation process assessing whether our training was observed as effective in teaching certain behaviors for our coaches.

### OSTE Implementation

**Fig. 1:**
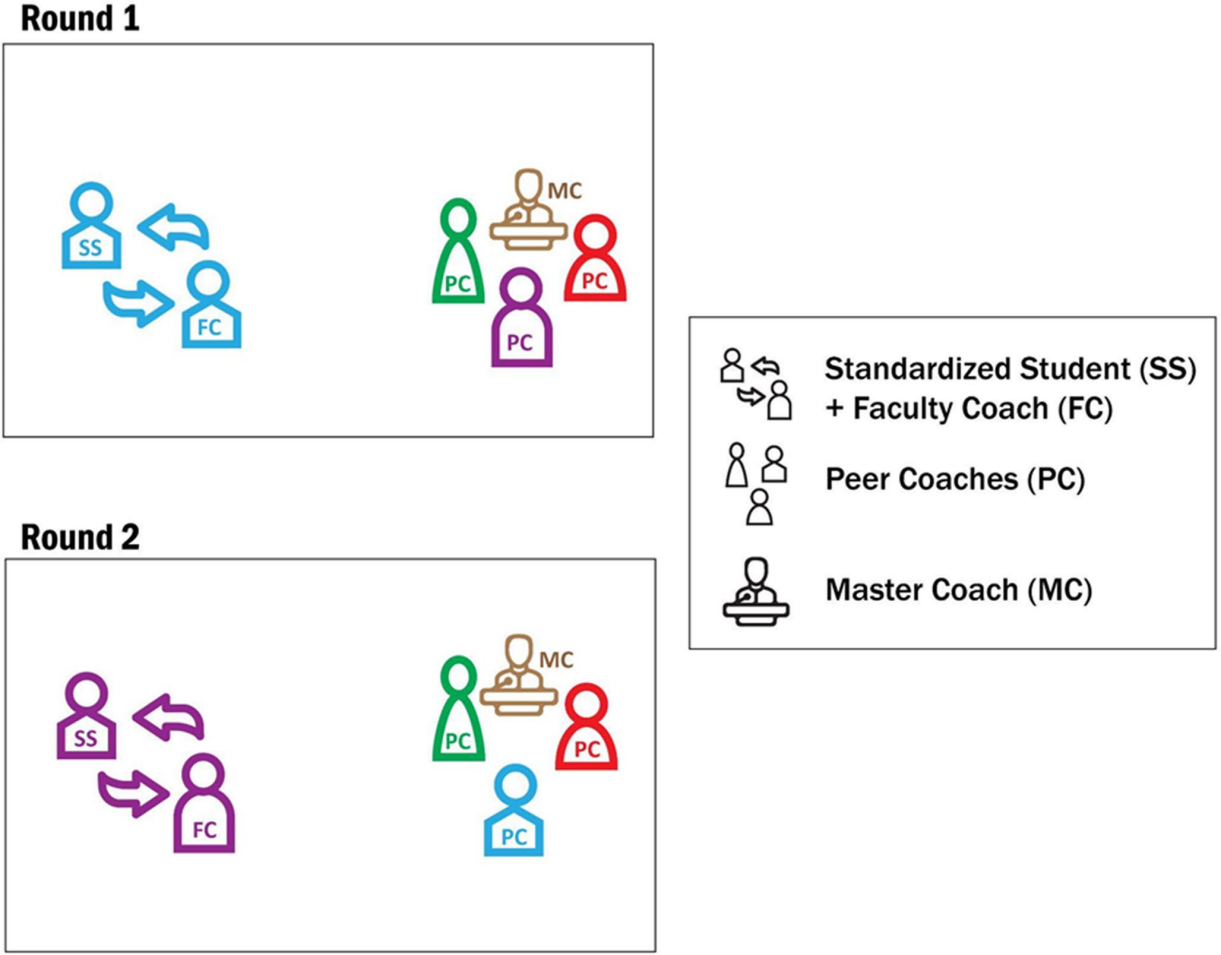
Depiction of the movement of the OSTE for different rounds. The faculty coach coaches a different standardized student while the other faculty coaches and master coach observe. Once the coaching session time is up, the faculty coach self-assesses their performance, the standardized student provides feedback and then the other faculty coaches and master coach provide feedback. Then the next standardized student enters and a new faculty coach coaches that student

A total of 53 faculty participated in the OSTE across three cohorts (22 in 2022, 19 in 2023, and 12 in 2024). Each cohort completed a single four-hour OSTE session consisting of four standardized coaching cases. In each case, faculty engaged in a 20-minute coaching encounter followed by 15 min of structured feedback. Within each cohort, faculty rotated through the four cases such that each faculty member served as the coach once and observed three additional cases. This structure ensured that all participants experienced the full set of four coaching scenarios while receiving feedback from standardized students, peers, and a master coach. Feedback began with a self-assessment by the faculty longitudinal coach in the simulation. Subsequently, and in the following order, further feedback continued from the standardized student, peer feedback from the other three longitudinal coaches observing the simulation, and all information was synthesized and gaps in feedback were filled in by the master coach faculty in the room. The standardized student could leave after delivering their feedback to allow for time movement from room to room or stay for the duration of feedback delivery if time allowed. The standardized students moved from room to room and the faculty stayed. (See Fig. 1). In total, faculty saw four realistic coaching scenarios to prepare them for their years as a longitudinal coach aligning with four of the 12 required coaching sessions in our program. See Table 1 for the objectives of each case and alignment within the program.

**Table 1:** Cases and objectives

Case #	Aligned Coaching Session	Objectives
1	4 (after 2 modules)	1. Academic success and resources 2. Transitioning study skills and volume of information
2	7 (after all pre-clinical education)	1. Burnout in medical education and personal wellness 2. Preparation for the next phase of education
3	8 (after 1–2 clinical rotations)	1. Professional identity formation conflicts 2. Profession reconsideration
4	10 (after all required clinical rotations and thinking about residency selection)	1. Handling the performer who is excelling 2. How to encourage continued performance and keep motivation

### Data Collection

Faculty participating in the simulation were given a handwritten feedback form to complete the day of the simulation. In the second and third year- faculty were given a follow-up survey via email months after starting their work as a coach. These surveys included information regarding the entirety of the training program for faculty development, not just this simulation. All master coaches completed the dichotomous checklist on our participants year two and three during the simulation on handwritten forms and returned to the administrator overseeing the simulation for tabulation. The surveys were designed based on Kirkpatrick’s levels to evaluate educational programs [32, 33]. All survey questions and checklist items are reported using descriptive statistics. Since there were no specific exploratory open-ended questions, formal thematic analysis was not performed. Comments were reviewed simply for trends. Open-ended comments from the day-of simulation forms and follow-up surveys were reviewed using a simple trend-identification approach. Because the forms did not include exploratory qualitative prompts and the volume of comments was small, we did not conduct a formal qualitative analysis.

## Results

Fifty-three faculty members from various professional backgrounds (See Table 2) participated in the four case OSTE. Qualitative comments from both the simulation day and follow-up surveys were reviewed for recurring trends. Participants consistently emphasized the usefulness of hands-on practice, the realism of the standardized student portrayals, and the value of receiving multi-source feedback. Table 3 presents representative quotes that illustrate these commonly expressed sentiments. For the second and third cohort (thirty-one participants) the five observable behaviors occurred with varied frequency (See Table 4). 77.4% were able to get at least one goal or agreement on what to talk about or action item articulated from the standardized student, 67.7% were able to elicit at least one barrier the student had to performance/action planned, 90.3% listened to the student’s perspective without interrupting, 77.4% avoided “why” questions, or closed ended (yes/no) questions, and 83.8% did minimal telling or advising even when asked for such items from the standardized student.

**Table 2:** Number of participants and professional backgrounds by cohort

	Date start	Total number in OSTE	# specialties
1 st cohort	Aug 22	22	Anesthesiology − 1 Emergency Medicine − 2 Family Medicine − 7 Gynecologic Surgery & Obstetrics − 3 Medicine - Internal Medicine − 1 Medicine - Pulmonology − 1 Pediatrics − 4 Psychiatry − 1 Radiology − 1 Surgery − 1
2nd cohort	Aug 23	19	Emergency Medicine − 1 Family Medicine − 1 Gynecologic Surgery & Obstetrics − 2 Medicine - Cardiology − 1 Medicine - Internal Medicine − 2 Medicine - PhD − 1 Medicine - Pulmonology − 1 Neurology − 1 Pediatrics − 5 Preventative Medicine & Biostatistics − 1 Psychiatry − 1 Surgery − 2
3rd cohort	Aug 24	12	Anesthesiology − 1 Emergency Medicine − 1 Family Medicine − 2 Medicine - Nephrology − 1 Pathology − 2 Pediatrics − 2 Preventive Medicine & Biostatistics − 2 Radiology − 1

**Table 3:** Representative participant comments by thematic trend

Thematic Trend	Representative Participant Quotes
Value of hands-on practice and structured feedback	“This was a great opportunity to actually practice the skills we’ve been talking about.” “By far the most helpful. The chance to practice & get feedback was incredibly valuable.” “Helpful (but painful) process. I learned a lot from the other faculty.”
Realism and quality of the standardized student encounters	“Role players were excellent; session leader was excellent with clear feedback.” “Well organized. Clear instructions. High yield learning activity.” “The students were exceptional in their portrayals.”
Increased confidence and comfort with coaching	“It made me feel much more comfortable going into my first few sessions.” “Avoidance of ‘why’ questions…has helped both personally and professionally.” “Let the student develop the solution. Don’t be so fast to ‘jump in’ with your solution.”

**Table 4:** Checklist of observed behaviors during the simulation

Question	Yes	No
The coach was able to get at least one goal/agreement on what to talk about/action item articulated from the standardized student.	24 (77.4%)	7 (22.6%)
The coach was able to elicit at least one barrier the student has to performance/action planned.	21 (67.7%)	10 (32.3%)
The coach listened to the student’s perspective without interrupting.	28 (90.3%)	3 (9.7%)
The coach avoided “why” questions, or closed ended (yes/no) questions.	24 (77.4%)	7 (22.6%)
The coach did minimal telling or advising even when asked for such items from the standardized student.	26 (83.8%)	5 (16.2%)

Several questions were asked in the follow-up survey months after the faculty development OSTE occurred. Thirty-seven people responded to the follow-up survey (69.8%). Of those responding 89.3% strongly agreed or agreed with the statement “I was able to engage in coaching to elicit my student’s goals and create plans,” 83.8% strongly agreed or agreed with the statement “The simulation aided in my preparation for coaching.,” and 78.4% strongly agreed or agreed with the statement “I found the simulation realistic regarding the student situations and perspectives.”

## Discussion

This study describes the development and implementation of an Objective Structured Teaching Encounter (OSTE) to enhance faculty coaching skills within a UME longitudinal coaching program. To our knowledge, this is the first published study applying the OSTE approach specifically to faculty development in medical education coaching, and one of the largest published with more than 50 participants [5]. While simulation-based education is well-established in clinical skill training and teaching development, its use in coaching, particularly for cultivating “coach-like” behaviors, remains underexplored.

Simulation has been increasingly incorporated into faculty development to strengthen communication, feedback, and teaching skills. Beyond OSTEs, approaches such as role-play, standardized learner interactions, and structured feedback workshops have demonstrated effectiveness in supporting faculty skill development and reflective practice [14, 26]. Simulation has also been used to help learners and educators navigate professional identity formation and real-time clinical feedback, highlighting its versatility in promoting interpersonal and teaching competencies [20, 21]. These modalities share core design elements with the OSTE, including psychological safety, deliberate practice, and guided reflection, which reinforces the relevance of simulation-based strategies for coaching development. Situating our OSTE within this broader simulation literature adds another example of multiple forms of simulation to contribute to scalable, practice-oriented faculty development.

By using a well-established simulation method, OSTE provided a rigorous, structured approach to coaching skill development, offering faculty a controlled environment to practice and refine their techniques while also allowing faculty to make mistakes in a safe, simulated situation. Due to the unique, small group style, faculty received personalized feedback for use in future encounters as well as tips and tricks for best practices from the master coach. Additionally, the three simulations observed allowed for more learning in an actual encounter and peer feedback. Peer feedback validated concerns of the new coaches while providing an opportunity to create community.

Unlike traditional, formal coaching certification programs, this initiative focused on fostering foundational, “coach-like” behaviors (such as active listening, avoiding assumptions, and promoting learner-driven dialogue) without requiring extensive and expensive external training [34]. The additional challenge for educators is adapting from advising to coaching mindset by way of question asking versus solution telling. The use of standardized students allowed for consistency across cases, ensuring faculty were exposed to common coaching challenges to practice new coaching skills in a realistic but low risk setting [5]. These standardized cases also allowed the faculty to experience a range of potential coachee problems and scenarios, thus limiting the surprise they may face and allowing the coaching program to highlight typical scenarios that could be encountered in future real-world cases intentionally to apply the coaching skills.

Simulation in coaching development offers unique advantages. It provides faculty with exposure to adaptive challenges, such as navigating coachee uncertainty or addressing nonverbal cues that may hinder rapport [5, 13]. The ability to observe oneself through feedback from standardized students, peers, and master coaches encourages reflection on personal coaching styles, including nonverbal communication and conversational dynamics which sheds light on yet unknown ways in which the faculty’s communication and style could jeopardize trust with future students and relationships. This aligns with adult learning principles, supporting adaptability and growth in real-time [35]. The fishbowl model [36, 37] embedded within the OSTE further maximized learning by allowing faculty to observe diverse approaches to similar coaching scenarios, even within a relatively short timeframe.

### Strengths

Key strengths of this initiative include its multi-cohort implementation, attention to best practices in OSTE development, use of immediate and post-surveys to capture participant feedback, and incorporation of observable behaviors aligned with established standards. Using three cohorts adds to the strength as results were consistent across all cohorts. Decisions made regarding the development of the simulation, training of the standardized students, and feedback structure aligned with the published recommendations on the use of OSTEs. Multipronged participant evaluation including immediate and remote surveys gave a more robust evaluation beyond Kirkpatrick’s level 1. The incorporation of the checklist of observable behaviors added to the objective assessment of the training. Even though there were only five items, these aligned with accepted coach competencies [38]. We had to change these into behaviors that could be observed in a short session. For example, “listens effectively” is an accepted competency which is aligned with our checklist item to “listens without interrupting.”

The diverse faculty population and involvement of master coaches— already familiar with the curriculum—allowed for contextualized, relevant feedback without necessitating additional external coaches or coaching certifications. The combination of self-assessment, standardized student (the coachee), peer feedback, and master coach input provided participants with a rich, multi-faceted understanding of their coaching competencies. Additionally, the low-cost, embedded nature of the program supports institutional sustainability and return on investment, offering an alternative to more resource-intensive faculty development models. Finally, coaches indicated (months following the training) that they continued to use core elements of coaching in their daily life, showing the long-term retainment of key skills and abilities for coaches.

### Limitations

This study was conducted at a single, specialty institution, which may limit generalizability. The number of observable behaviors was intentionally focused, which, while practical, may not capture the full spectrum of coaching competencies. Survey data relied on self-reports and were limited in scope due to confidentiality considerations, which constrained our ability to link individual performance to outcomes. Also, while immediate post-intervention feedback was positive, longer-term impacts on faculty coaching practices were not assessed. Additionally, because the study relied primarily on descriptive statistics, our ability to draw inferences about the change in coaching behaviors is limited. The findings should be interpreted as exploratory rather than evaluative. Finally, while Table 4 includes binary frequencies, inferential statistics were not conducted because the checklist was designed for formative evaluation rather than hypothesis testing.

### Future Directions

Future work could explore whether simulation-based coaching development translates into sustained behavioral change and improved learner outcomes. Expanding the scope to include multi-institutional studies could enhance generalizability, scenarios and cases of the standardized student. There is also potential to script common coaching challenges, such as addressing medical knowledge gaps or professional identity concerns, to standardize training across programs. Exploring the role of coaching skills beyond formal coaching sessions, such as their application in clinical teaching, leadership, and even personal interactions, could also further highlight the value of cultivating a coach-like mindset across professional domains. The use of fishbowl training, whether with standardized cases or peer observation, presents an efficient method to expose faculty to diverse coaching strategies within limited timeframes. Finally, assessing outcomes beyond participant satisfaction (Kirkpatrick Level 1) and observation (Level 2) will be important. Evaluating changes in self-assessed coaching abilities (Level 3) and potential longitudinal impacts on learner development would provide a more comprehensive understanding of the return on investment for simulation-based coaching development.

## Conclusions

This study demonstrates that an OSTE can serve as an effective and scalable approach to developing foundational coaching skills in undergraduate medical education faculty. When provided with opportunities to practice coaching behaviors in realistic scenarios, receive structured feedback from peers and master coaches, and reflect on performance, participants reported greater confidence and sustained use of coaching strategies in their work. Additionally, institutions can create scenarios that align with their individual coaching programs. Unlike formal certification programs, this model offers a low-cost, embedded method that aligns with institutional goals for faculty development and competency-based education. As coaching becomes a more integral part of medical training, simulation-based approaches like this OSTE offer a practical solution to equip faculty with the tools needed to support learner growth, adaptability, and reflection across diverse settings.

## Declarations

### Disclaimer

The views expressed in this paper are those of the authors and do not reflect the official policy of the Uniformed Services University of the Health Sciences, the Department of the Army, Navy, Air Force, Department of War, or the U.S. Government.
